# Too drained to help? How work overload fuels psychological strain and why proactive personality functions as a buffer

**DOI:** 10.3389/fpsyg.2025.1705661

**Published:** 2026-01-08

**Authors:** Zesheng Wei, Juling Wang, Quanyi Gao

**Affiliations:** 1 Department of Human Resource Management, School of Economics and Management, Xi’an Technological University, Xi’an, China; 2 Department of Organization Management, School of Management, Xi’an Jiaotong University, Xi’an, China

**Keywords:** helping behavior, JD-R model, proactive personality, psychological strain, work overload

## Abstract

By integrating the job demands-resources (JD-R) model with the proactivity literature, this study investigates the psychological mechanism and boundary condition that link work overload to helping behavior. We test our hypotheses using a four-wave, multi-source dataset from 366 employee-supervisor dyads in a manufacturing firm in China. The findings show that work overload is positively related to psychological strain, which in turn reduces helping behavior. Moreover, proactive personality functions as a critical personal resource; it buffers the detrimental effect of work overload on psychological strain, thereby weakening the indirect negative effect of work overload on helping behavior. These results contribute to the literature by uncovering the resource-depletion pathway between job demands and helping behavior, while highlighting proactive personality as an agentic, behavioral trait that drives employees to managing heavy workloads and sustain resource-sensitive helping behavior in demanding work environments.

## Introduction

Employees today face a ubiquitous dual challenge in their daily work (Lin et al., 2019; Kumar et al., 2021). Across many organizational settings, escalating work demands and overwhelming role responsibilities have become commonplace as firms strive to maintain competitiveness in dynamic environments (Jensen et al., 2013; Laurence et al., 2016; Kumar et al., 2021). In parallel, the growing emphasis on collaborative work requires employees to devote increasing time and energy to helping colleagues (Bolino et al., 2013; He et al., 2023; Kim et al., 2023), with some reports indicating that collaborative tasks can consume up to 80% of the workday (Cross et al., 2016). This places employees in a relentless bind: they must fulfill their own demanding work roles while simultaneously serving as a resource for helping others. This dilemma holds profound implications for both individual well-being and organizational effectiveness.

While extensive research has explored the detrimental consequences of work overload as well as the benefits and costs of helping behavior (Lin et al., 2019; Chen et al., 2020; Koopman et al., 2020; Ganster et al., 2023), the relationship between these two constructs remains puzzling (Eatough et al., 2011; Bachrach et al., 2024). The job stress literature clearly documents that work overload depletes employees’ finite mental and physical resources, thereby impairing performance (Cavanaugh et al., 2000; Pluta and Rudawska, 2021; Zeschke et al., 2025). Meanwhile, helping behavior is increasingly recognized as a double-edged sword. Although lauded for its benefits to recipients and organizations (Koopman et al., 2016; Harari et al., 2022), it is also a resource-consuming activity that can lead to emotional exhaustion, work–family conflict, and even career stagnation for helpers (Bergeron et al., 2013; Lin et al., 2019; Lin et al., 2020; Hong et al., 2025). This inherent resource conflict has yielded inconsistent findings: some studies find that work overload diminishes helping behavior, while others report non-significant or even positive relationships (Chhabra, 2016; Jenkins et al., 2016; Lee et al., 2019). Consequently, the critical question of *how* the psychological toll of work overload shapes employees’ capacity to help remains largely unanswered, leaving the underlying mechanisms and boundary conditions underexplored.

**Figure 1:**
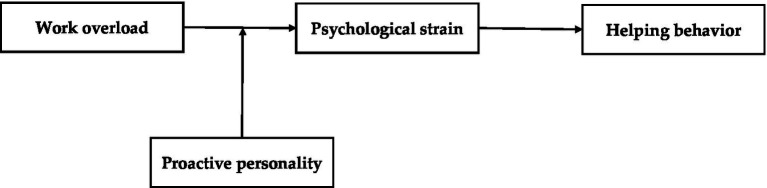
Hypothesized research model.

To resolve this ambiguity, we draw on the job demands-resources (JD-R) model (Demerouti et al., 2001) to propose a moderated mediation model. Specifically, we posit that psychological strain serves as the central mediating mechanism. According to the JD-R model, excessive job demands trigger a health-impairment process that depletes employees’ resources and undermines well-being (Bakker and Demerouti, 2017; Bakker et al., 2023). In this context, strain directly consumes the personal resources necessary for engaging in discretionary helping behavior. However, not all employees succumb to this pressure equally. We further propose that proactive personality functions as a critical buffer that mitigates the positive effects of work overload on psychological strain. As an agentic, behavioral trait-like resource, proactive personality drives employees to actively alter their environment and reduce the burden created by heavy demands (Parker et al., 2006; Cangiano et al., 2019). By engaging in such proactive efforts in changing demanding environments, we hypothesize that proactive personality weakens the positive relationship between work overload and psychological strain, thereby preserving employees’ capacity to help colleagues. Our proposed research model is depicted in Figure 1.

This study makes three contributions to the literature. First, we extend the JD-R model by positioning proactive personality as a stable, trait-like personal resource that serves as a critical buffer in the health-impairment process. Although proactivity-related concepts, such as job crafting, are well-established within the JD-R model (Tims et al., 2012), existing research primarily views proactive personality merely as an antecedent of proactive behavior (Bakker et al., 2012). We shift the focus to a more nuanced question: rather than simply asking whether proactive individuals shape their jobs, we examine how their dispositional tendency to take initiative equips them with the inherent resilience under high job demands (Loi et al., 2016; Chu and Chou, 2024). We posit that proactive personality acts as an internal engine of initiative and control, enabling employees to conserve psychological resources when confronted with depleting work demands (Parker et al., 2006; Cangiano et al., 2019). This perspective offers a new theoretical explanation for why some employees can endure work overload while retaining the psychological capacity to helping others.

Second, we clarify the puzzling relationship between work overload and helping behavior by pinpointing both the underlying mechanism and a critical boundary condition. Prior research has struggled to explain why overloaded employees exhibit varying levels of helping behavior (Eatough et al., 2011; Bachrach et al., 2024). Our findings show that work overload diminishes helping behavior indirectly through heightened psychological strain, consistent with the JD–R model’s health-impairment pathway (Bakker and Demerouti, 2017). Furthermore, by revealing proactive personality as a crucial moderator, we clarify for *whom* work overload is most detrimental and, consequently, most likely to erode helping behavior.

Third, we enrich the JD–R model by advancing a resource-based understanding of helping behavior. Our findings indicate that helping is not merely a function of prosocial motivation or resource supportive conditions (Bakker et al., 2004), but is also a resource-consuming behavioral outcome that is sensitive to the health-impairment process. Our findings show that work overload triggers a resource-depletion process that manifests as psychological strain, thereby restricting the resources available for helping. This perspective aligns with the “dark side of helping” literature (Bergeron et al., 2013) by reframing helping as a context-contingent behavior that fluctuates with resource availability. By demonstrating how and when high work overload stifles employees’ capacity to help, we extend the JD-R model by offering a clearer explanation for why demanding work environments often yield declines in voluntary, pro-organizational helping behavior.

### Theory and hypothesis development

#### Work overload and helping behavior: the mediating role of psychological strain

According to the JD-R model, work overload is conceptualized as a prototypical job demand—defined as the perception of having “too much work and too little time” (Harvey et al., 2003, p. 306). These demands require sustained physical or mental effort, thereby draining employees’ physical, emotional, and cognitive resources (Bakker and Demerouti, 2017; Bakker et al., 2023). This resource depletion impairs employees’ ability to meet work requirements and triggers a health-impairment process that manifests as psychological strain. Meanwhile, helping behavior—defined as discretionary actions aimed at assisting coworkers and supporting organizational functioning (Organ, 1997; Organ et al., 2006)—is increasingly recognized as a resource-sensitive activity (Bolino et al., 2013; Koopman et al., 2020; Harari et al., 2022). Although helping benefits recipients and organizations, it also consumes the helper’s time, energy, and emotional resources, potentially leading to emotional exhaustion and reduced personal effectiveness (Bolino and Turnley, 2005; Halbesleben et al., 2009; Lin et al., 2019). According to the JD-R model, when employees’ resources are depleted by high demands, they become less capable of enacting these discretionary behaviors (Bakker and Demerouti, 2017). This creates a dilemma for overloaded employees: despite a willingness or perceived obligation to assist others, their diminished resource pool may limit their capacity to do so. However, existing literature provides an inconsistent empirical finding for the relationship between work overload and helping behavior (Eatough et al., 2011; Bachrach et al., 2024).

To resolve this inconsistency, we draw on the JD-R model to bring in psychological strain as a key mediator underlying. Psychological strain reflects adverse internal psychological states, including job-related anxiety and depression, resulting from prolonged exposure to high demands (Sprigg et al., 2007). The JD-R model posits that coping with excessive demands like work overload requires compensatory effort, consequently depleting employees’ personal resources (Bakker and Demerouti, 2007, 2017; Bakker et al., 2023). Consistent with this view, prior research has demonstrated that work stressors are robust predictors of undesirable psychological and physical outcomes, including stress-related illness and impaired well-being (Karasek, 1979; Nixon et al., 2011; Bowling et al., 2015).

We further propose that the psychological strain resulting from work overload inhibits helping behavior. Helping is not merely a passive act; it requires a coordinated series of resource-intensive actions, such as diverting attention from one’s own work, identifying colleagues in need, listening to their problems, and determining appropriate forms of assistance (Bachrach et al., 2024). These activities demand not only time and energy but also considerable cognitive effort and emotional regulation, which can interfere with the helper’s ability to complete their own work (Nielsen et al., 2012; Podsakoff et al., 2009). Grounded in the foundational work on JD-R model (e.g., Demerouti et al., 2001), high job demands first consume employees’ finite energetic and cognitive resources. This resource depletion manifests psychologically as strain. Once employees enter this strained, resource-depleted psychological state, their capacity for discretionary, resource-consuming behaviors—such as helping colleagues—are significantly impaired. Therefore, employees experiencing psychological strain are less likely to help others, as they must conserve their remaining resources available to cope with their core duties. Take together, we propose that work overload triggers a resource-depletion process that manifests as psychological strain, which in turn reduces helping behavior:

*H1*: Psychological strain mediates the relationship between work overload and helping behavior.

### The moderating role of proactive personality

Proactive personality is a trait-like disposition characterized by the enduring tendency to actively initiate change (Bateman and Crant, 1993; Crant, 2000). Compared to their passive counterparts, proactive individuals are more adept at identifying opportunities, taking initiative, and persevering through obstacles to achieve meaningful changes (Bateman and Crant, 1993; Parker et al., 2010). Crucially, they are predisposed to interpret stressors, such as work overload, not merely as threats but as challenges or opportunities for mastery and improvement (Loi et al., 2016; Chen et al., 2021; Chu and Chou, 2024).

The JD-R model posits that personal resources—such as self-efficacy, self-esteem, and optimism—reflect an individual’s perceived ability to influence and control their environments. These personal resources function as a buffer, mitigating the detrimental impact of job demands on strain (Demerouti et al., 2001; Bakker and Demerouti, 2017). Building on this theoretical perspective, we identify proactive personality as a critical personal resource that buffers the positive effect of work overload on psychological strain. Proactive individuals possess a variety of resource-enhancing traits, including role breadth self-efficacy, flexible role orientation, and positive state affectivity, which fuel engagement in change-oriented proactive behaviors (Bateman and Crant, 1993; Parker et al., 2006; Hu and Liao, 2024). When facing high workload, proactive employees are less likely to succumb to pressure and more likely to take initiative, develop and enlarge social networks, proactively crafting their jobs, and handling challenging work situations (Thompson, 2005; Parker et al., 2010; Zhang et al., 2021). These active coping efforts allow them to mitigate resource loss and replenish depleted energy, thereby reducing psychological strain (Wei et al., 2021). Moreover, proactive individuals possess a strong sense of agency and competence, which bolsters their belief in their ability to overcome situational constraints (Parker et al., 2006; Cangiano et al., 2019). As a result, proactive individuals tend to maintain a deeper reservoir of personal resources, weakening the positive impact of work overload on psychological strain.

In contrast, less proactive individuals tend to adapt passively to adverse work situations rather than attempting to shape them (Bateman and Crant, 1993; Crant, 2000). Lacking the inclination to engage in resource-generating behaviors, they are less equipped to manage the effect of work overload. Without the buffering effect of proactive personality, these employees bear the full weight of job demands, rendering them more susceptible to psychological strain (Bakker and Demerouti, 2017; Wei et al., 2021). Therefore, we argue that proactive personality attenuates the positive relationship between work overload and psychological strain. Based on these arguments, we propose the following moderating hypothesis:

*H2*: Proactive personality moderates the positive relationship between work overload and psychological strain, such that this relationship is weaker when proactive personality is higher and stronger when proactive personality is lower.

### Moderated mediation effects

Integrating the mediating role of psychological strain with the moderating effect of proactive personality, we propose a moderated mediation model. We posit that the indirect effect of work overload on helping behaviors through psychological strain is contingent upon an individual’s level of proactive personality. Specifically, employees high in proactive personality are better equipped to neutralize the detrimental effects of work overload. Because they actively engage in coping behaviors to manage high job demands and possess substantial personal resources, they can buffer the health-impairment process caused by work overload (Bakker and Demerouti, 2017; Chen et al., 2021; Wei et al., 2021). As a result, they experience low psychological strain even when facing overload. By preserving cognitive and emotional resources, these proactive individuals tend to retain the energetic capacity necessary to engage in discretionary, resource-consuming behaviors such as helping behavior (Halbesleben et al., 2009; Sprigg et al., 2007). In contrast, individuals low in proactive personality are more likely to passively adapt to high workload conditions rather than actively managing the associated stressors (Bateman and Crant, 1993). Lacking the buffering benefits of proactivity, they are more susceptible to the direct transfer of work overload into high psychological strain. This elevated strain can deplete their resource reservoir, leaving them with insufficient resources to devote to helping others. Take together, we propose the following moderated mediation hypothesis:

*H3*: Proactive personality moderates the indirect negative effect of work overload on helping behavior through psychological strain, such that this indirect relationship is weaker when proactive personality is higher and stronger when proactive personality is lower.

## Method

### Sample and procedure

Data were collected from a large manufacturing company located in western China. The company engaged in the production and marketing of semiconductor components, liquid crystal display screens and other electronic products and components. Through on-site visits and interviews with the Director of Human Resources and Administration, we were informed that front-line production employees and their immediate managers not only need to complete heavy and repetitive work but also require frequent collaboration. With support from the Human Resource department, we contacted 507 full-time employees and their 89 immediate supervisors to participate in our study. Participants were recruited from various manufacturing functions, including manufacturing, detection, front-end control, packaging, and testing. Participants were informed that participation was voluntary, and their responses would be strictly confidential and used solely for research purposes.

To reduce concerns regarding common method bias, we employed a four-wave, multi-source design to temporally separate the predictors, mediator, and outcome variables with an approximately three-week interval (Podsakoff et al., 2012). At Time 1, employee participants were asked to report their demographics and to rate proactive personality. A total of 463 employees completed this survey, yielding a response rate of 91.3%. At Time 2, the 463 employees who completed the Time 1 survey were invited to rate the perception of work overload. A total of 415 employees completed the second survey, resulting in a response rate of 89.6%. At Time 3, we invited 415 subordinates who completed the Time 2 survey to rate psychological strain. A total of 376 employees completed the third survey, resulting in a response rate of 90.6%. At Time 4, the immediate supervisors of the 376 employees who completed the Time 3 survey were invited to evaluate the helping behaviors of their subordinates via an online survey. After matching the responses across the four phases and dropping cases with irregular or incomplete responses, the final sample contained 366 sets of employee-supervisor matched cases nested with 82 supervisors (response rate: 92.1%).

Among the 366 employee participants, 64.21% were male, the average age was 27.09 years (*SD* = 3.60), average organizational tenure was 4.65 years (*SD* = 2.37), and about 39.07% had an associate college degree or higher.

### Measures

We followed standard back-translation procedure (Brislin, 1986) to translate the original items written in English into Chinese. Unless otherwise noted, items were rated on a 5-point Likert scale ranging from 1 (*never*) to 5 (*always*).

#### Proactive personality

We used 10 items from Seibert et al. (1999) to measure proactive personality. Responses were based on a 7-point Likert scale ranging from 1 = “strongly disagree” to 7 = “strongly agree.” A sample item is “I am constantly on the lookout for new ways to improve my life” (*α* = 0.91).

#### Work overload

We measured the perception of work overload using four items developed by Brown and Benson (2005). A sample item is “my job leaves me with very little time to get everything done” (*α* = 0.65). Following the procedure of Hayes and Coutts (2020), the McDonald’s Omega value of work overload is 0.66 and the factor loadings of the four items in the scale are 0.449, 0.477, 0.719, and 0.625, respectively.

#### Psychological strain

We adopted six items from Sprigg et al. (2007) to measure psychological strain. This variable included two sub-dimensions: job-related anxiety and job-related depression. Sample items are “I feel worried” (job-related anxiety) and “I feel miserable” (job-related depression). The Cronbach’s alpha was 0.89.

#### Helping behavior

We measured helping behavior using three items shortened by De jong et al. (2007), originated from Settoon and Mossholder (2002). Responses were based on a 5-point Likert scale ranging from 1 = “strongly disagree” to 5 = “strongly agree.” A sample item is “This subordinator assists other with heavy workloads even though it is not part of the job” (*α* = 0.88).

#### Control variables

We controlled for the demographic variables of age (in years), gender (0 = male; 1 = female), organizational tenure (in years), and education level (high school or below = 1; associate/vocational = 2; bachelor = 3; master or above = 4) following previous studies (e.g., Laurence et al., 2016; Kumar et al., 2021; Bachrach et al., 2024). All results remained consistent after removing all control variables in the analysis.

### Analytical strategies

Each supervisor rated the helping behavior of one or more subordinates, giving rise to a nested data structure. We employed the ANALYSIS commands TYPE = COMPLEX and ESTIMATOR = MLR in Mplus 8.4 to estimate the models (Muthén and Muthén 1998/2015). To test our hypotheses, we first ran a path model to test mediating effects (Hypothesis 1), and then ran an integrated path model, including two regression models, to test our moderation model (Hypothesis 2) and moderated mediation model (Hypothesis 3).

In the first regression model, we adopted the Monte Carlo resampling method with 20,000 repetitions to calculate the 95% confidence interval (CI) to test the significance of indirect effects. In the second regression model, we regressed psychological strain on demographics, work overload, proactive personality, and the interaction term between work overload and proactive personality based on the process of mean-centered (Aiken and West, 1991). In the third regression model, we regressed helping behavior on demographics, work overload, and psychological strain. We then adopted the Monte Carlo resampling method to calculate the 95% confidence interval (CI) through repeatedly performing random sampling to test the significance of the conditional indirect effects (Preacher and Selig, 2012).

## Results

### Confirmatory factor analysis

We conducted a series of confirmatory factor analyses (CFAs) to assess the distinctiveness validity of the four focal variables, namely, work overload, psychological strain, proactive personality, and helping behavior by using the item parceling approach (Landis et al., 2000). As our sample size was not large, we used item parceling to obtain reliable estimates. Otherwise, the model would exceed the recommended parameter to sample size ratio for estimation (1:5; Bentler and Chou, 1987). We sequentially averaged the combined items with the highest and lowest loadings and created three parcels for each latent variable (Landis et al., 2000). The CFA results are shown in Table 1. As expected, the baseline four-factor model fit the data well (χ^2^ = 73.93, *df* = 48, *p <* 0.01, RMSEA = 0.04, CFI = 0.98, TLI = 0.98, SRMR = 0.04), and was better than (a) the three-factor model combining work overload and psychological strain into one common factor (∆χ^2^ *=* 102.16, ∆*df* = 3, *p <* 0.01, RMSEA = 0.08, CFI = 0.92, TLI = 0.90, SRMR = 0.07), (b) the two-factor model combining work overload, proactive personality, and psychological strain into one common factor (∆χ^2^ *=* 664.68, ∆*df* = 5, *p <* 0.01, RMSEA = 0.19, CFI = 0.58, TLI = 0.48, SRMR = 0.17), and (c) the one-factor model combining the four variables (*∆*χ^2^ = 1138.85, *∆df* = 6, *p* < 0.01, RMSEA = 0.24, CFI = 0.29, TLI = 0.13, SRMR = 0.22). Table 2 presents the descriptive statistics, correlations, and reliability of the focal variables.

**Table 1:** Results of confirmatory factor analyses.

Model	χ2	*df*	∆χ2	RMSEA	CFI	TLI	SRMR
Four-factor model (the baseline model)	73.93	48		0.04	0.98	0.98	0.04
Three-factor model: combine work overload and psychological strain	176.09	51	102.16**	0.08	0.92	0.90	0.07
Two factor model: combine work overload, proactive personality, and psychological strain	738.61	53	664.68**	0.19	0.58	0.48	0.17
One factor model	1212.78	54	1138.85**	0.24	0.29	0.13	0.22

*N* = 366. RMSEA = Root Mean Square Error of Approximation; CFI = Comparative Fit Index; TLI = Tucker-Lewis Index; and SRMR = Standardized Root-Mean-Square Residual. ^**^p < 0.01.

**Table 2:** Descriptive statistics, correlations, and reliabilities.

Variables	Mean	*SD*	1	2	3	4	5	6	7	8
1. Sex	0.64	0.48								
2. Age	27.09	3.60	0.00							
3. Degree	2.30	0.70	−0.66^**^	−0.03						
4. Organizational tenure	4.65	2.37	−0.07	0.74^**^	0.10					
5. Work overload	2.61	0.70	−0.10	−0.03	0.13^*^	0.02	(0.65)			
6. Proactive personality	5.85	0.88	−0.12^*^	0.22^**^	0.06	0.28^**^	−0.03	(0.91)		
7. Psychological strain	1.61	0.63	−0.12^*^	−0.13^*^	0.21^**^	−0.09	0.20^**^	−0.12^*^	(0.89)	
8. Helping behavior	4.32	0.73	−0.06	0.06	0.02	0.09	−0.18^**^	0.05	−0.24^**^	(0.88)

N = 366. Cronbach’s alphas are shown on the diagonal in parentheses. ^*^p < 0.05, ^**^p < 0.01.

### Test of mediation effects

We first conducted a path model to test the mediation effect in Table 3. This mediation model was satisfactory (χ^2^(0) = 0, *p < 0.01*, RMSEA = 0.00, CFI = 1.00, TLI = 1.00, SRMR = 0.00). As seen in Table 3, work overload was positively related to psychological strain (*β* = 0.16, p < 0.01). Meanwhile, psychological strain was negatively related to helping behavior (*β* = −0.29, p < 0.01). Table 4 shows the results of mediation effects. As expected, work overload had a negative indirect effect on helping behavior through psychological strain (indirect effect = −0.041, 95% CI = [−0.090, −0.013]), lending support to Hypothesis 1.

**Table 3:** Regression results of path model analyses.

Predictor	Psychological strain		Helping behavior	
	Model 1	Model 2	Model 3	Model 4
Sex	−0.06(0.10)	0.05(0.10)	−0.11(0.12)	−0.12(0.12)
Age	−0.01(0.02)	−0.01(0.01)	−0.01(0.02)	−0.01(0.02)
Degree	0.20^**^(0.07)	0.19^**^(0.07)	0.01(0.09)	0.02(0.09)
Organizational tenure	−0.02(0.02)	−0.01(0.02)	0.03(0.03)	0.03(0.03)
Work overload	0.16^**^(0.05)	0.24^**^(0.05)		−0.16^**^(0.05)
Proactive personality		−0.07(0.04)		
Work overload × Proactive personality		−0.18^**^(0.05)		
Psychological strain			−0.29^**^(0.08)	−0.26^**^(0.08)

*N* = 366. Standard errors are shown in parentheses. Unstandardized regression coefficients are reported. ^*^p < 0.05, ^**^p < 0.01.

**Table 4:** Results for mediation effect and moderated mediation effect tests.

Indirect effect	Estimate	SE	95%CI	
			Lower	Upper
Work overload → Psychological strain → Helping behavior	−0.041	0.019	−0.090	−0.013

Conditional indirect effect	Moderator	Estimate	*SE*	95% CI	
				Lower	Upper
Work overload → Psychological strain → Helping behavior	High PP	−0.021	0.015	−0.0614	0.0050
	Low PP	−0.101	0.042	−0.1922	−0.0304

*N* = 366. PP = Proactive personality. SE = Standard error. CI = Confidence interval. Results of CI are obtained through Monte Carlo resampling method.

### Test of moderation effects

**Figure 2:**
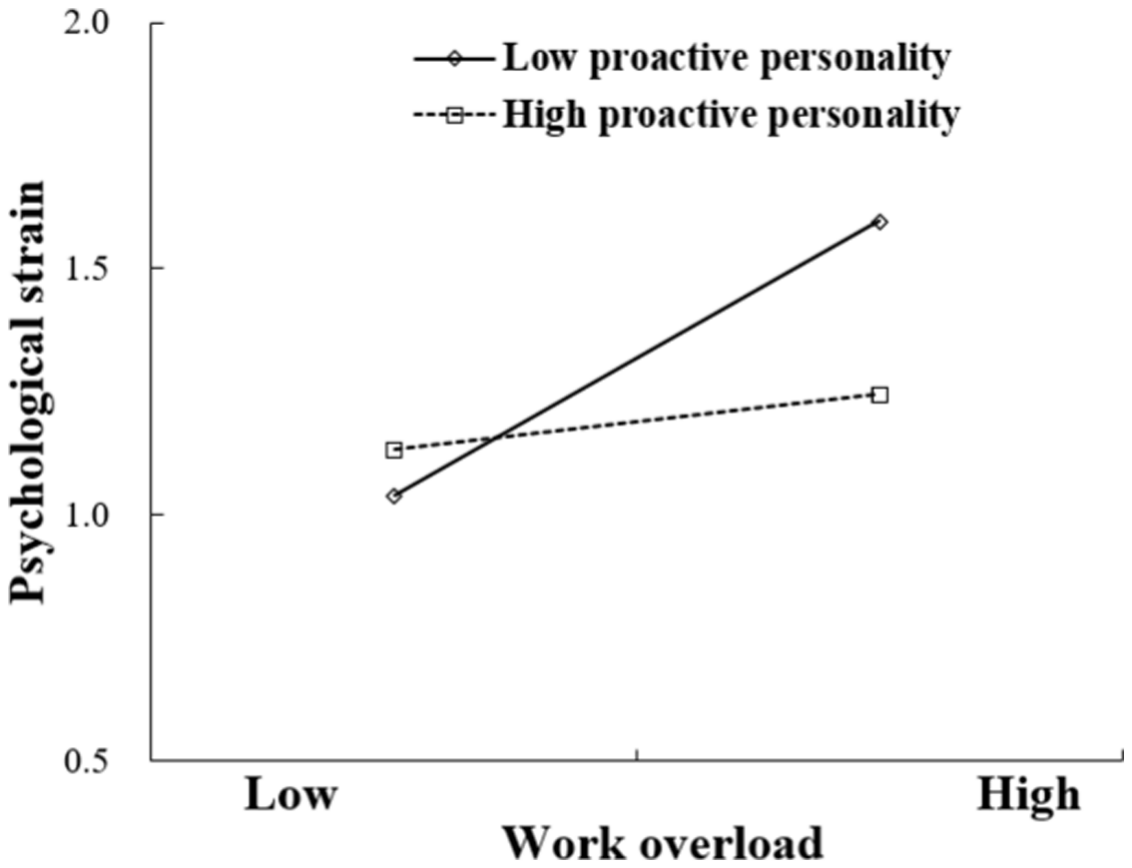
Simple slope for the interaction effect of work overload and proactive personality on psychological strain.

Model 2 in Table 3 presents the results for the moderating effects of proactive personality. This moderation model was satisfactory [χ^2^(0) = 0, *p <* 0.01, RMSEA = 0.00, CFI = 1.00, TLI = 1.00, SRMR = 0.00]. The regression results revealed that the interactive term between work overload and proactive personality was negatively related to psychological strain (*β* = −0.18, *p <* 0.01). Figure 2 presents the interaction pattern at high and low levels of proactive personality, defined as 1 SD above and below the mean value, respectively. The relationship between work overload and psychological strain was non-significant at high levels of proactive personality (+1 SD; slope = 0.08, SE = 0.05, *p >* 0.05, 95% CI = [−0.010, 0.171]) but was positive and significant at low levels of proactive personality (−1 SD; slope = 0.40, SE = 0.09, *p <* 0.01, 95% CI = [0.230, 0.563]). These results lend support to Hypothesis 2.

### Test of moderated mediation effects

We further conducted an additional path model to test the moderated mediation effect by adding the effects of proactive personality and the interaction between work overload and proactive personality on the mediator to the mediation path model. The rest of the specification in the model was the same as that in the mediation path model. This model demonstrated an adequate fit [χ^2^(11) = 56.64, *p <* 0.01, RMSEA = 0.00, CFI = 1.00, TLI = 1.00, SRMR = 0.00]. To examine whether adding the interaction term improved model fit, we compared our baseline moderated mediation path model with an alternative model in which the interaction effect was constrained as zero [χ^2^(15) = 71.12, *p <* 0.01, RMSEA = 0.08, CFI = 0.86, TLI = 0.32, SRMR = 0.04]. A chi-square difference test of the nested models using the Satorra-Bentler scaled chi-square (Satorra and Bentler, 2010) was significant (Δχ2 = 11.32, Δ*df* = 4, *p < 0.05*), suggesting that the model with the interaction effect was better.

To examine the moderated mediation hypothesis, we tested the conditional indirect effects of work overload on helping behavior through psychological strain at high and low levels of proactive personality. The model fit index of the integrated moderated mediation model was satisfactory, RMSEA = 0.00, CFI = 1.00, TLI = 1.00, SRMR = 0.00. As presented in Table 4, the indirect effect was significant when proactive personality was low (indirect effect = −0.101, 95% CI = [−0.1922, −0.0304]), but it became non-significant when proactive personality was high (indirect effect = −0.021, 95% CI = [−0.0614, 0.0050]). Thus, Hypothesis 3 was supported.

## Discussion

By integrating the JD-R model with the proactivity literature, this study proposed and tested a moderated mediation model to examine how work overload affects helping behavior through psychological strain, contingent upon proactive personality. The findings revealed that work overload exerted a negative indirect effect on helping behavior through enhanced psychological strain. Furthermore, our results showed that proactive personality weakens the positive relationship between work overload and psychological strain, thereby preserving the capacity for helping behavior. Therefore, the study provides a nuanced understanding of the psychological processes and individual differences that shape employee responses to job demands, offering meaningful contributions to the literatures on occupational stress, helping behavior, and personality at work.

### Theoretical implications

This research contributes to literature in several important ways. First, we advance theory by establishing a novel role for proactive personality within the JD-R model. While prior research has primarily viewed proactive personality as an antecedent of proactive behaviors, such as job crafting (e.g., Bakker et al., 2012), we position it as a crucial trait-like personal resource that buffers the health-impairment process central to the JD-R model. Our findings show that work overload triggers a resource-depletion cycle—heightening strain and reducing helping behavior—but proactive personality interrupts this negative cycle. Unlike optimism (expecting good outcomes) and self-efficacy (believing in one’ s capability), which are cognitive resources that primarily enable individuals to endure or reframe stressful situations (Bakker and Demerouti, 2007; Xanthopoulou et al., 2009), proactive personality is an agentic, behavioral trait (Crant, 2000). It drives employees to actively alter their environment to reduce the burden of work overload (Bakker and Demerouti, 2017; Bakker et al., 2023). By engaging in proactive efforts in changing demanding environments, proactive individuals do not simply withstand work overload; they actively restructure it. Therefore, we demonstrate how proactive personality functions as a protective shield that preserves the psychological resources necessary for discretionary, extra-role behavior.

Second, our study resolves specific inconsistencies in the literature regarding the relationship between work overload and helping behavior. Prior research has yielded mixed findings, with some studies showing a negative link while others finding no significant relationship (e.g., Chhabra, 2016; Kumar et al., 2021; Lee et al., 2019). This creates a theoretical puzzle: why do some overloaded employees withdraw from helping while others do not? Our findings provide clarity by demonstrating that the negative effect of overload on helping is not direct, but is mediated by psychological strain. This mechanism aligns with the JD-R model’s health-impairment pathway (Bakker and Demerouti, 2017), demonstrating that employees refrain from resource-consuming helping behaviors, specifically when they experience the resource depletion associated with strain (Halbesleben and Bowler, 2007). Furthermore, the moderating role of proactive personality clarifies for *whom* this process occurs: the negative pathway is significant primarily for low-proactive employees. This resolves previous ambiguities by identifying proactive personality as the boundary condition that determines whether work overload translates into psychological strain and subsequent helping withdrawal.

Third, we offer a more nuanced expansion of the JD-R model by positioning helping behavior as a significant outcome of the health-impairment process. While the JD-R model has extensively explored outcomes such as in-role performance and burnout (Bakker and Demerouti, 2007), helping behavior is typically associated with the motivational “gain spiral.” In line with the “dark side of helping” literature (Bergeron et al., 2013), our findings demonstrate that helping is not solely a function of motivation (Bakker et al., 2004), but is contingent on the preservation of personal resources. In doing so, we integrate helping behavior into the JD-R model’s “loss spiral,” moving beyond the predominant focus on its role in the “gain spiral” of resource accumulation. By explicitly linking helping behavior to the health-impairment process, our study provides a novel and comprehensive explanation for how, why, and when work overload prevents employees from engaging in extra-role, discretionary helping behavior, which is invaluable for organizations.

### Practical implications

Our research offers important practical implications for organizations and managers. First, organizations should adopt a balanced view on the relationship between work overload and helping behavior, recognizing that excessive workload not only compromises employee well-being but also depletes their capacity to engage in discretionary helping. Our results showed that work overload hindered helping behavior by enhancing psychological strain. Therefore, organizations should prioritize designing manageable workloads, enhancing structural job resources (e.g., autonomy, supervisory support), and cultivating a psychologically healthy work environment (LaMontagne et al., 2007). Such organizational-level interventions reduce strain at its source and help sustain a workplace climate that encourages voluntary cooperation and mutual support among employees.

Second, our research highlights the importance of supporting employees’ psychological well-being, particularly for those in high-demand roles where helping behavior may be compromised. Beyond establishing healthy organizational-level work conditions, organizations can implement evidence-based individual-level interventions to further alleviate strain (Bakker and Demerouti, 2017). Programs such as resilience training, mindfulness programs, and cognitive-behavioral workshops should be provided to strengthen employees’ coping capacities and mitigate the harmful psychological effects of work overload. These interventions not only improve individual well-being but also increase the likelihood that employees will sustain prosocial behaviors even under demanding work conditions.

Third, our findings suggest that proactive personality, as a key personal resource, can buffer the detrimental effect of work overload on psychological stain, thereby preserving employees’ capacity for helping behavior. Employees high in proactive personality are less likely to suffer strain under high demanding conditions. Accordingly, organizations should consider incorporating assessments of proactive traits in recruitment processes, while also fostering proactivity through training and development programs. Interventions designed to cultivate initiative-taking, problem-solving, and self-leadership may help build a more resilient and prosocial organizational culture.

### Limitations and directions for future research

Our research has several limitations that need to be addressed in future research. First, although we collected data from multiple sources across multiple waves, our cross-sectional design could not examine the causality among the studied variables. We encourage the adoption of longitudinal or experimental studies to rigorously test causal directions. In addition, our data were collected from employees within a single manufacturing firm in China. As such, the results may not fully capture how these mechanisms operate in other types of organizations, industries, or cultural contexts, which encourages us to interpret the findings and related conclusions very cautiously. Future research should benefit from examining whether the observed relationships hold across diverse job contexts—such as service-oriented or knowledge-intensive work—and in different cultural settings.

Second, the reliability values of work overload scale, while acceptable for exploratory purposes (*α* = 0.65), was relatively modest. This may partly stem from the small number of items or variation in how work overload is perceived across individuals (Li et al., 2011; Li et al., 2018). In addition, the average inter-item correlation is reasonable (0.32). Future research should seek to replicate our proposed research model using other plausible measures of work overload.

Third, our study examines only the initiating stage of the loss cycle in the JD-R model, showing how work overload triggers psychological strain and reduces helping behavior. Future research should extend this work by investigating the reciprocal pathway whereby reduced helping and heightened strain may, in turn, intensify work overload further. To capture these dynamic processes, scholars are encouraged to employ cross-lagged panels or other longitudinal designs that can distinguish between initiating and reinforcing mechanisms. Such work would offer a more comprehensive test of the cyclical nature of resource dynamics proposed in the JD–R model.

Finally, while personal resources (such as self-efficacy, organizational-based self-esteem, and optimism) are theorized to be in mitigating the resource-consuming process (Xanthopoulou et al., 2009; Loi et al., 2020), we focused exclusively on the moderating role of proactive personality in the health-impairment process, and the indirect effect of high job demands on helping behavior. To enrich the research on the health-impairment process and the loss spiral embedded in JD-R model, future research should identify other job resources or personal resources that may mitigate the initiating pathway of the loss cycle.

## Conclusion

This study advances scholarly understanding of the psychological mechanisms and boundary conditions underlying the relationship between work overload and helping behavior. By integrating the JD-R model with research on proactive personality, we demonstrate that psychological strain serves as a critical mediator through which work overload reduces helping behavior. Importantly, our findings reveal that this health-impairment pathway is buffered by proactive personality, highlighting its role as a personal resource that enables employees to protect their resources when facing work overload. These findings underscore the complex interplay between job demands, individual well-being, and helping behavior. As organizations increasingly rely on interpersonal collaboration in high-demand environments, understanding how and for whom work overload impairs helping behavior is crucial. Our study offers actionable insights for researchers and practitioners seeking to foster healthier, more supportive, and more effective workplaces.

## Data Availability

The raw data supporting the conclusions of this article will be made available by the authors, without undue reservation.
